# Re-Examining Rotavirus Innate Immune Evasion: Potential Applications of the Reverse Genetics System

**DOI:** 10.1128/mbio.01308-22

**Published:** 2022-06-14

**Authors:** Avan Antia, Amanda N. Pinski, Siyuan Ding

**Affiliations:** a Department of Molecular Microbiology, Washington University School of Medicine, St. Louis, Missouri, USA; Duke University; Ohio State University

**Keywords:** innate immunity, reverse genetic analysis, rotavirus

## Abstract

Rotaviruses represent one of the most successful pathogens in the world, with high infectivity and efficient transmission between the young of many animal species, including humans. To overcome host defenses, rotaviruses have evolved a plethora of strategies to effectively evade the innate immune response, establish initial infection in the small intestine, produce progeny, and shed into the environment. Previously, studying the roles and relative contributions of specific rotaviral factors in innate immune evasion had been challenging without a plasmid-only reverse genetics system. Although still in its infancy, current reverse genetics technology will help address important research questions regarding rotavirus innate immune evasion, host range restriction, and viral pathogenesis. In this review, we summarize the current knowledge about the antiviral host innate immune defense mechanisms, countermeasures of rotavirus-encoded factors, and strategies to better understand these interactions using the rotavirus reverse genetics system.

## INTRODUCTION TO ROTAVIRUS

Group A rotaviruses (RVs) are among the most common causes of diarrhea-induced morbidity and mortality in infants and children under the age of 5 worldwide (1). RV typically causes severe acute dehydrating gastroenteritis, with about 1 in 65 infections leading to hospitalization and 1 in 300 infections leading to death (2). Although the global implementation of RV vaccines, which began in 2006, has reduced disease severity and deaths, RV infections remain a major cause of pediatric mortality today (3). For instance, RV caused 128,500 deaths and over 258 million episodes of diarrhea in children under 5 years old in 2016 alone (1).

RV is a member of the *Reoviridae* family and consists of 11 double-stranded (ds)RNA segments encapsidated within a triple-layered protein shell. The RV genome encodes six structural proteins (VP1 to VP4, VP6, and VP7) and six nonstructural proteins (NSP1 to NSP6). Each gene segment encodes a single gene product, with the exception of gene segment 11, which encodes NSP5 and NSP6 in overlapping open reading frames (4, 5). The structural proteins assemble to form the viral capsid and spike structure, while the nonstructural proteins play roles in viral replication, virulence, and host immune antagonism (6). The strain-specific variation of RV gene sequences and protein functions is a major contributor to host-range restriction (HRR), a phenomenon in which RVs replicate in and best antagonize the host species from which they are originally discovered and isolated.

All segmented RNA viruses, including RVs, can undergo genetic reassortment, which is the exchange of gene segments between strains. This occurs when two RV strains of the same genogroup coinfect a single cell, as the segments of one are likely to be packaged into the virion of the other. Isolation of the resulting RV reassortants has been instrumental in studying RV innate immune evasion, allowing researchers to examine the strain-specific impact of a particular RV segment. Prior to the development of a highly efficient plasmid-only reverse genetics (RG) system, researchers relied on natural selection to isolate these reassortants (7). Natural RV reassortants also provide the basis of the current and widely used Rotateq (RV5) vaccine (8). Today, a plasmid-only RV RG system enables the generation of purposefully modified reassortants, fluorescent and bioluminescent reporter viruses, and viruses with point mutations, truncations, domain swaps, and epitope tags in specific proteins, empowering the study of individual RV proteins and yielding insights into the genetic and molecular basis of immune evasion and HRR in unprecedented ways, as discussed below.

In this review, we discuss the arms race between RV and host innate immune pathways and how these interactions contribute to HRR. We will discuss both what is known and not known about RV-mediated host antagonism, and how the remaining gaps can be filled using the novel plasmid-only RV RG system. By clarifying specific determinants of innate immune antagonism with RG, we can ultimately create safe and effective next-generation vaccines, and further our understanding of the biology of segmented RNA viruses and virus-host interactions.

## PRINCIPLES OF INNATE IMMUNE RESPONSE TO dsRNA VIRUSES

The innate immune response to infection with a dsRNA virus, as for any other pathogen, involves two major steps: sensing viral infection and responding appropriately (9, 10). Sensing of viral dsRNA occurs through cytoplasmic RIG-I-like receptors (RLRs; e.g., RIG-I, MDA5) and/or endosomal Toll-like receptors (TLRs; e.g., TLR-3, -7, and -8) (11, 12). Specifically, RV sensing occurs primarily via cytosolic RIG-I and MDA-5 (11, 12), endosomal TLRs 3 and 7 (13, 14), and the Nlrp9b inflammasome (15). These receptors activate adaptor proteins, like mitochondrial antiviral-signaling protein (MAVS) for RLRs and MyD88, TRAF6, and/or TRIF for TLRs, which then recruit specific transcription factors, such as interferon regulatory factors (IRFs), AP-1, and NF-κB, to drive type I/III interferon (IFN) and cytokine expression. Recognition of closely related mammalian orthoreovirus and bluetongue virus also requires RIG-I, MDA5, and MAVS (16, 17). Secreted IFNs bind to their respective receptors—IFNAR1/2 for type I IFNs and IFNRL1/IL10RB for type III IFNs—on both infected and uninfected bystander cells. Upon receptor engagement, IFN receptor-associated JAKs are activated by autophosphorylation, which in turn phosphorylate STAT1 and STAT2. Activated STAT1, STAT2, and IRF9 form an ISGF3 transcription factor complex, which initiates transcription of a large number of interferon-stimulated genes (ISGs) that amplify the initial antiviral response and restrict the viral life cycle. Detailed reviews regarding antiviral innate immunity are available (9, 10).

## HOST RANGE RESTRICTION OF ROTAVIRUS INFECTION

The host range of a virus is defined as the breadth of species in which a virus can infect and replicate. RV can cause disease in the young of many mammals, including humans, pigs, cattle, mice, and primates (5). Host range restriction thereby describes the constraints on viral replication in a specific host, which are determined by viral and host factors. Several early studies documented this phenomenon using murine and porcine models, and although attempts have been made to identify responsible viral proteins, no consensus has yet been reached (18–21). Feng et al. (22) demonstrated that homologous murine RV EW strain replicates 1,000- to 10,000-fold more efficiently in the mouse intestine than the heterologous simian RV RRV strain does, and implicated NSP1 as a necessary, but not sufficient, determinant of this HRR. Phylogenetic analyses show that NSP1 varies among species and plays a role in HRR, as demonstrated in murine models (22–24). VP3, VP4, NSP2, and NSP3 also appear to support robust RV EW replication in the mouse gut (22). Saxena et al. (25) demonstrated that select human RV strains (e.g., Ito G3P[8]) infect human small intestinal enteroids 100- to 1000-fold more efficiently than a simian RV RRV strain. Similarly, a virulent porcine RV G9P[13] strain replicates better in porcine small intestinal enteroids than a human RV Wa strain (26). The viral determinants of robust RV infection of enteroids of respective species remain to be identified.

Antagonism of host innate immunity by RV proteins likely contributes to HRR. In other words, an RV strain is adapted to the innate immune system of its native species (i.e., homologous host) but not to that of another species (i.e., heterologous host). For example, the replication of heterologous simian RRV is significantly enhanced in mice lacking type I/II IFNs or STAT1, whereas that of a homologous strain is not (27). While *STAT1* knockout mice only shed 5-fold more murine RV than wild-type mice, *STAT1* knockout mice shed 100-fold more heterologous simian RRV (28). *In vitro* studies also show that RRV replication is enhanced in human colonic epithelial HT-29 cells lacking MAVS, while the replication of the homologous human RV Wa strain is unaltered (29). Different homologous RV strains are variably affected by IFN signaling deficiencies (27). A single-cell analysis of RV-infected intestinal epithelial cells (IECs) uncovered heterogeneity in the transcription of IFNs and ISGs among different cell subsets. Results indicated that type III IFN may play a role in restricting heterologous RRV replication, but not homologous EW replication, in mice (30). Collectively, these studies demonstrate that host innate immunity influences RV replication and HRR, yet the multifactorial determinants of the host restriction have yet to be clarified.

## FENDING OFF THE HOST INNATE IMMUNE SYSTEM: ROTAVIRAL FACTORS AND THEIR HOST TARGETS

With a relatively small genome, many RV-encoded proteins have evolved multiple purposes in both the viral life cycle and host defense antagonism. In the following sections, we discuss key RV innate immune antagonists, including NSP1, VP3, NSP2, and NSP3, and delve into the mechanisms by which these factors interact with their host targets to promote RV infection.

### NSP1: the anti-host “Swiss army knife.”

**FIG 1:**
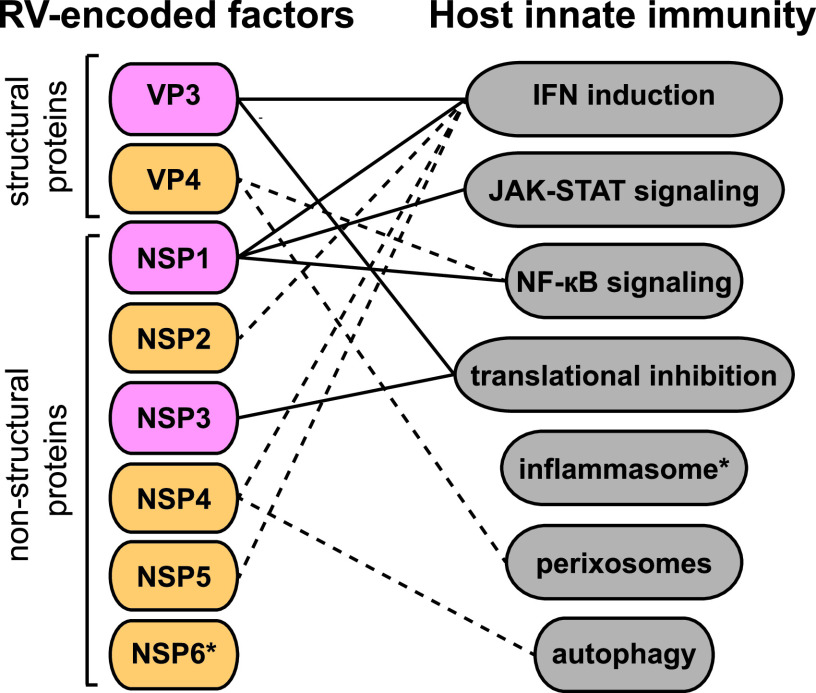
Complex interactions of the arms race between rotavirus (RV)-encoded viral factors and host innate immune signaling pathways. Solid lines: proven inhibition; dotted lines: putative inhibition; pink: known RV factors that antagonize distinct pathways; orange: possible RV factors that inhibit innate immunity. Asterisks indicate viral factors and host signaling pathways whose respective targets are yet to be identified via the reverse genetics (RG) system.

NSP1 is the least conserved of the RV proteins. NSP1 targets a number of host proteins and antagonizes host innate immunity (Fig. 1), yet it is dispensable for *in vitro* replication (31). Encoded by RV gene segment 5, NSP1 is an approximately 57-kDa protein with a conserved N-terminal RING domain and variable C-terminal domain (32). The RING domain implicates NSP1 as having potential viral E3 ubiquitin ligase activity, although this has not been experimentally proven (33–35).

**(a) IFN regulatory factors (IRFs).** The IRF family of transcription factors includes nine members (36), and different IRFs are triggered by distinct stimuli, yet induce overlapping transcriptional programs (37). All IRFs have an N-terminal DNA binding domain that recognizes IRSE-like DNA sequences and a C-terminal domain that typically contains a nuclear export sequence and an autoinhibitory domain (36). IRFs 3, 5, and 7 contain an IRF association domain (IAD) near the C terminus that mediates IRF dimerization into an active transcriptional complex (36). IRF9 has an IAD-like region that promotes interactions with the phosphorylated STAT1/STAT2 heterodimer to form ISGF3. Dimerization is not required for IRF9 function (36).

Several IRFs are targeted by RV NSP1. NSP1 was initially shown to interact with IRF3 in a yeast two-hybrid assay (38). Later studies showed that NSP1s from many bovine, murine, and simian RV strains also interact with the IADs of IRFs 3, 5, and 7 and the IAD-like region of IRF9 to induce proteosome-dependent degradation of these IRFs (36, 39, 40). NSP1-IRF interactions are mediated through the C terminus of NSP1 via a pLxIS motif, where p is a hydrophilic residue and x is any amino acid (41). The conserved pLxIS motif is also present in multiple host proteins, such as MAVS, STING, and TRIF (41, 42). Thus, RV exploits this conserved pLxIS motif for immune evasion by associating with and degrading IRFs, thereby blocking downstream signaling (41).

Strain-dependent NSP1-IRF interactions highlight HRR. Generally, NSP1s from non-human strains degrade IRFs 1, 3, 5, and 7, whereas NSP1s from most human strains primarily degrade IRFs 5 and 7 (43). For example, bovine UK strain replication is IFN-restricted in mouse embryonic fibroblasts because, unlike murine EW and simian RRV strains, UK NSP1 cannot degrade murine IRF3 (44). In contrast, some bovine NSP1s can degrade human IRF3 (45). *In vitro* studies have found that NSP1 proteins from human, porcine, bovine, and simian backgrounds effectively caused IRF1 degradation-independent and -dependent inhibition (36, 45, 46). For instance, infection of African green monkey MA104 cells with homologous simian RRV also results in an early (12 hours post-infection) decrease in IRF1 protein levels (29). In contrast to IRF3 and IRF7, which induce type I and III IFN production downstream of mitochondrial MAVS, IRF1 seems to be primarily activated by MAVS residing on peroxisomal membranes and is key for type III IFN production (47, 48). Since type III IFNs only act on the small subset of cells expressing IFNLR1 (e.g., intestinal epithelial cells) to effectively control intestinal viral infection *in vivo*, inhibition of IRF1 highlights a key role for epithelial cells—rather than immune cells—in early RV control (30, 48–52). Whether the same mechanism is shared with other viral antagonists of IRFs, such as classical swine fever virus or bovine viral diarrhea virus N-terminal proteases, has not been examined (53–55).

**(b) β-TrCP.** NF-κB signaling is critical for host antiviral, proinflammatory responses, but is inhibited by RV in an HRR-manner. NF-κB activation stimulates phosphorylation of IκB by an IκB kinase, which creates a phosphodegeron motif to mark IκB for degradation by β-TrCP, a subunit of the Skp1-Cul1-F-box ubiquitin ligase complex (56, 57). NF-κB then translocates to the nucleus, where it initiates transcription of various cytokines. RV NSP1 targets β-TrCP for proteasomal degradation to inhibit NF-κB signaling (56, 57). Of note, human, bovine, and porcine NSP1s mediate β-TrCP degradation while other animal NSP1s preferentially target IRFs, likely contributing to HRR. For example, NSP1s from human Wa and ST3 strains and porcine OSU strains degrade β-TrCP, while NSP1 from the bovine NCDV strain targets IRF3 (57). A phosphodegeron-like motif (DSGXS) in the NSP1 C terminus evidently serves as a decoy substrate for host β-TrCP recognition (58). One study demonstrated that genetic depletion or chemical inhibition of host ubiquitin ligase subunits Cul3 and the RING-box protein Rbx1 abrogates β-TrCP degradation (56). Of note, a different study showed the depletion of Cul3 using small interfering RNA (siRNA) does not hinder NSP1-mediated degradation of β-TrCP, suggesting that degradation is independent of NSP1 association with the host ubiquitin ligases (46). The discrepancy in results may arise from the use of different human RV strains (e.g., WI61 and DS-1 versus Wa and ST3), inconsistent siRNA knockdown efficiency, and the use of different cell lines (MA104 versus HEK293 cells).

**(c) STAT1.** STAT1 is critical for amplifying the initial IFN-induced antiviral response. Single-cell transcriptional analysis of infected murine small intestinal epithelium showed that RV strain EW prevents ISG expression in both infected and bystander IECs, despite robust IFN production by gut hematopoietic cells (30). This suggests that RV inhibits the effects of hematopoietic cell-derived IFN on infected and bystander cells. Holloway et al. (59, 60) observed that RV infection prevents the nuclear accumulation of activated STAT1 independent of NSP1. However, Sen et al. (61) identified NSP1 as a direct antagonist of both STAT1 phosphorylation and activation. These contradictory results may be explained by temporal differences between the studies—16 versus 12 h postinfection—potentially a short window of time for clear inhibitory capacity. On the other hand, there could be a combination of mechanisms that inhibit the downstream STAT signaling.

**(d) Other host factors.** There are numerous reports of interactions between NSP1 and other host proteins, which require further analysis by domain mapping and validation in the context of RV infection. TRAF2 acts as an adaptor protein downstream of various tumor necrosis factor α (TNF-α) superfamily members to mediate NF-κB signaling (62). The NSP1 C-terminal domain mediates TRAF2 proteosome-dependent degradation, blocking NF-κB signaling (63). NSP1 also targets different components of host apoptotic machinery. Bagchi et al. demonstrated that RV inhibits apoptosis in the host cell by activating the pro-survival PI3K/Akt pathway early in infection, and later demonstrated that early in infection, NSP1 targets p53, the classic cell stress-responsive tumor suppressor, for ubiquitin-mediated proteasomal degradation through a PI3K-independent mechanism (64, 65). NSP1 also seems to modulate host miRNA expression (66). By suppressing p53, NSP1 dampens the expression of host miRNA-29b, a suppressor of the epithelial-mesenchymal transition pathway, which can be triggered by various RNA viruses (66). Additional interactions with innate immune proteins are discussed in later sections.

### VP3, NSP2, and NSP3: a trio of partners in innate immune inhibition.

Host mRNAs have 5′ caps and 3′ poly(A) tails to mark them as “self,” thereby preventing cytosolic degradation by host RNases. To survive these conditions, the RV guanyl and methyl transferase VP3 caps the 5′ end of RV transcripts, while NSP3 binds a consensus sequence at the 3′ end of each RV transcript (67–69). In addition to its role in RNA capping, VP3 N terminus also targets MAVS for degradation in a strain-specific manner (Fig. 1), which dampens the type III IFN response *in vivo* (29). MAVS is also a target for cleavage and/or degradation by various RNA virus-encoded proteins, including hepatitis C virus NS3/4A (70), hepatitis A virus 3AB precursor (71), and SARS-CoV-2 ORF10 (72), although studies examining sequences and functional similarities among these virus-encoded MAVS antagonists have not yet been performed (29). VP3-mediated MAVS inhibition may also effect type I and III IFN production (29, 73). A recent study also determined that VP3 antagonizes the 2′,5′-oligoadenylate (2-5A) synthetase (OAS)-RNase L pathway (74, 75). This function of VP3 was discovered due to its structural homology to the mouse hepatitis virus ns2, another protein that inhibits 2-5A accumulation (76). The contribution of VP3 to HRR is not yet clearly defined; however, phylogenetic analysis of VP3 sequences from multiple RV strains identified 3 motifs which segregate with species, potentially allowing for species-specific protein-protein interactions and serving as a genetic basis for HRR (77, 78).

Competing with the host poly(A)-binding protein (PABP), NSP3 interacts with eIF4G, a component of the eIF4 ribosome recruitment complex necessary for translation (79). This protects viral RNAs from degradation, facilitating translation of viral mRNAs and inhibiting translation of host mRNAs (68, 69). NSP3 has also been found to influence the host unfolded protein response, a protective feedback mechanism that restricts translation in response to an accumulation of misfolded proteins in the endoplasmic reticulum (ER) (80). Simian RRV strain can upregulate various ER stress pathways at the transcriptional level; however, NSP3 simultaneously inhibits multiple stress response genes at the translational level (81). ER stress during RV infection may also result from inhibition of translation and accumulation of viral proteins, although a portion of viral antigens are sequestered in viroplasms, of which NSP2 is a key component (81). Although the mechanisms of NSP2 and NSP3 in innate antagonism and the identities of specific host protein targets are yet-to-be-defined (Fig. 1), these viral proteins add to the complexity of RV genes that contribute to HRR (22).

## RV STUDIES IN THE ERA OF REVERSE GENETICS

RG systems are powerful tools for determining the genotype-phenotype relationships of viral products in an otherwise constant genetic background. The recently developed plasmid-only RV RG systems allow the generation of reporter and mutant RVs that continue to provide critical insight into host-virus interactions. Such modified RVs were difficult to study prior to RG (29, 82, 83). For instance, determining the role of NSP1 in RV replication relied on comparing RV strains with naturally occurring mutations, deletions, and truncations rather than targeted alteration of specific amino acids (84–87). Plasmid-only RV RG systems will also facilitate the rational design and development of next-generation RV vaccine candidates with targeted attenuation.

### Brief history of plasmid-only RV RG systems.

RG systems require the transfection of DNA plasmids encoding the entire or individual segments of the viral genome into a recipient cell line and subsequent recovery of viable viral progeny (88–90). Early RV RG systems relied on helper viruses (e.g., vaccinia virus, human RV KU strain) and neutralizing antibody-based selection or dual selection of temperature-sensitive mutants (88, 91–95). This contrasted with the relatively efficient plasmid-only RG systems that existed for other segmented dsRNA (e.g., birnavirus, orthoreovirus, orbivirus) (96, 97) and single-stranded RNA (e.g., influenza virus, poliovirus, hepatitis C virus) (98–101) viruses. A long-awaited, plasmid-only RG system for simian RV SA11 strain was described in 2017 by Kanai et al. (87). The system was subsequently optimized and adapted by other groups to improve recovery of additional recombinant RV strains (102–104).

The RG system developed by Kanai et al. utilizes 11 plasmids each encoding a segment of the simian RV SA11 genome, three helper plasmids, and a bacteriophage T7 RNA polymerase-expressing BHK-21 cell line for transfection. The helper plasmids encode a fusion-associated small transmembrane protein from Nelson Bay orthoreovirus to facilitate viral spread, and the two subunits of the vaccinia virus capping enzyme to stabilize viral RNA and ensure translation (105, 106). The Kanai system was successfully used to recover human RV Odelia and KU strains (107, 108). Providing NSP2 and NSP5 plasmids at 3-fold greater levels than any other viral gene was subsequently shown to enhance recovery, likely by facilitating early viroplasm formation (107, 109–111). The RG system developed by Sánchez-Tacuba et al., which capitalizes on a plasmid encoding a fusion protein consisting of T7 polymerase and the African swine fever virus NP868R capping enzyme, as well as an IFN-insensitive MA104 cell line, has been used to recover hard-to-culture RV strains, including simian RRV and human CDC-9; a recombinant RRV expressing EGFP; a murine RV reassortment D6/2-like virus (104); a murine RV lacking NSP1 expression (112); and a murine RV encoding a Nano-Luciferase bioluminescent reporter (113). Extensive reviews of the current plasmid-only RG systems are available elsewhere (7, 114–116).

### Leveraging RG to understand RV innate immune evasion.

**(a) RNA sensing and antagonism of type I and III IFN signaling.** Upon entry into the cell, RLR-sensing of RV dsRNA activates MAVS (116, 117). Previous studies have demonstrated that RIG-I and MDA5 are independently essential for antiviral responses to RV *in vitro*, suggesting that RV generates RNA species recognized by both receptors, which are degraded by NSP1 (12, 118, 119). The endosomal dsRNA sensor TLR3 appears to have a negligible role in restricting RV *in vitro*, although the absence of TLR3 in infected mice increases viral shedding and histopathology and reduces the type I IFN response without inducing diarrhea (11, 12, 120, 121). Future studies are required to precisely probe dsRNA in intracellular compartments distinct from viroplasms and double-layered particles that shield RV from immune detection.

MAVS activation leads to IFN expression mediated by transcription factors IRF-1, -3, -5, and/or -7 (117). Future studies can leverage RG to determine the molecular basis for selective targeting by NSP1 and its connection to host cell species and tissue type, RV strain, and IAD sequence, as discussed in earlier sections. Domain swapping, site-directed mutagenesis, NSP1-deletion RVs, and monoreassortant RVs in an isogenic backbone will be particularly useful in addressing these questions. Fluorescently tagged NSP1 can clarify the spatiotemporal dynamics of NSP1 during infection in relation to known targets (IRF3, β-TrCP, STAT1, and others). For instance, whether NSP1 targets substrates for degradation or whether there are several NSP1 pools dedicated to each substrate remains to be tested.

RV NSP1 may also target IRF1, despite the obvious lack of an IAD (29, 45). The minimum sequence requirement for this inhibition and the connection to RV strains can be determined using the RG system. The RG system can also be used to investigate proteasome-mediated downregulation of surface type I/II/III IFN receptor expression *in vitro* and *in vivo* (122). Given that downregulation of IFN receptors is sufficient to prevent mortality from lethal endotoxin exposure in RV-infected mice, the determination of such mechanisms has relevance to other infectious diseases (122). It is important to note that the E3 ubiquitin ligase activity of NSP1 is still speculative (43, 123). Mutation of key catalytic residues in the N-terminal RING-finger domain using RG and evaluation of changes in polyubiquitination will facilitate the testing of this hypothesis.

With regard to VP3, refined mapping of the VP3-MAVS binding site will instruct site-directed mutagenesis to recover recombinant viruses without MAVS targeting ability. Such studies will help to determine the relative contributions of NSP1-mediated IRF degradation versus VP3-mediated MAVS inhibition in blocking IFN induction during RV infection (Fig. 1).

**(b) DNA sensing and antagonism of type I and III IFN signaling.** The cGAS-STING pathway canonically senses and responds to DNA viruses. Following the sensing of double-stranded DNA in the cytosol by cGAS, STING activates IRF3 and NF-κB to induce type I and III IFN expression (124). Interestingly, a number of RNA viruses, including enteric viruses like murine norovirus, are restricted by the cGAS-STING pathway (125). Many also encode cGAS and/or STING antagonists (126–128). Published work and a non-peer reviewed study show that activation of the cGAS-STING pathway may be due to leaked genomic or mitochondrial DNA, which may be integrally tied to a recently discovered, non-canonical role for cGAS in mediating DNA damage responses (129–131).

The role of cGAS-STING in RV pathogenesis has not been investigated; however, it is likely precisely controlled during RV infection to avoid triggering host innate immunity. Specifically, RV NSP4 is an ER-resident protein that triggers calcium flux from the ER, leading to the activation of store-operated calcium efflux by ion channel STIM1 at the ER-plasma membrane interface, subsequently inducing diarrhea (132). STIM1 typically associates with STING at the ER. However, activation permits the relocation of STING to the Golgi, where it rapidly responds to cyclic dinucleotides (133). Mislocalization of STING to the ER or Golgi via STIM1 dissociation enhances basal levels of IFN, which primes the antiviral response to DNA viruses and leads to autoinflammatory disease (133). A recent study determined that loss of cohesin complex member STAG2 increased DNA damage and activated a robust cGAS-STING-mediated antiviral response that restricted RV replication (131). RG system-based screening for candidate cGAS-STING antagonists will enable characterization of the spatial dynamics of nuclear or mitochondrial DNA damage and leakage events during RV infection.

**(c) Antagonism of NF-κB signaling.** NF-κB activation during RV infection leads to a cytokine signature distinct from that induced by type I/III IFN signaling (134, 135). However, RV infection, at least *in vivo*, is not characterized by a strong inflammatory status (135, 136). NSP1 antagonizes this pathway via proteasome-mediated degradation of β-TrCP in a strain-specific manner (43, 56, 57, 123). Other viral proteins may also be involved. The N-terminal VP8* fragment of VP4 contains a conserved PXQX(T/S) sequence, which typically mediates interactions between TNF receptors and TRAF adaptor proteins (137) that can lead to NF-κB activation. Human and simian VP8* interacts with TRAF2 to promote NF-κB activation in the absence of TNF or other stimuli (137). TNF-α treatment blunts viral replication *in vitro* (138). Whether the contradictory effects of NSP1 and VP8* on NF-κB signaling are mutually exclusive for a given RV strain has not been determined. Site-directed mutagenesis studies with the RG system should avoid conflicting defaults in viral entry with differences in TRAF2 activation (139).

Studies demonstrating direct protein-protein interactions between NSP1 or VP4 and a component of NF-κB signaling pathway, as well as increased ubiquitination of target proteins, are lacking or inconsistent. For instance, mass spectrometry-based screens determined that several members of the ubiquitin ligase complex, Cul1, Cul3, and Rbx1, are binding partners of NSP1 in a strain-specific manner (46, 56, 140). The roles of VP8*, NSP1, and other RV proteins in NF-κB signaling antagonism can now be accurately parsed using the RG system to understand protein localization, host binding partners, and critical interaction residues (Fig. 1).

**(d) Manipulation of host translation.** Like many viruses, RV inhibits host protein synthesis upon infection, thereby halting innate antiviral and stress responses and favoring the synthesis of viral proteins (141). The utility of this mechanism in evading the host response is questionable given that type I and III IFN can escape translational arrest (142). Nonetheless, several mechanisms may explain reduced protein synthesis in RV infection (143).

mRNA stability and decay. During infection, detection of dsRNA by OAS triggers production of 2-5A, activating RNase L to nonspecifically cleave host and viral transcripts to inhibit translation (144). Recent studies have shown that this can occur independent of IFN (142). VP3 2′-5′ phosphodiesterase activity cleaves 2-5A and disrupts the OAS-RNase L signaling axis (74). A recent study used the RV RG system to generate recombinant RVs with VP3 point mutations and demonstrated that VP3 phosphodiesterase activity is critical for *in vitro* and *in vivo* replication (145). However, this mechanism of translational inhibition may occur primarily in IECs. Future RG studies can dissect this tissue/cell specificity as well as the contribution of other VP3 activities, such as RNA-capping activity and disruption of OAS-RNase L signaling (67).

Translation. NSP3 binds to a conserved consensus sequence in the RV 3′ untranslated regions to facilitate translation (68, 69, 146–151). NSP3 expressed from a chimeric vaccinia virus in nonhuman primate kidney cells is sufficient to reduce host translation to levels comparable to those during RV infection (68, 152, 153). NSP3 interactions with eIFG4G and ZC3H7B also induce nuclear accumulation of PABP to prevent host mRNA export to the cytoplasm (68, 154). Disrupting these interactions does not alter viral replication kinetics or translation (150, 155–158). The molecular mechanisms used by the host to avoid degradation of IFN during translational arrest possibly parallel the strategies used by RV (142).

Protein-protein interactome analysis, in conjunction with the RG system, will be instrumental for identifying host and viral factors that specifically facilitate replication (159). Swapping NSP3 RNA-binding domains between RV strains and other viruses (e.g., flavivirus NS2A, vaccinia virus E3L) will reveal species- and virus-specific mechanisms of translational antagonisms (160, 161). RG can also be used to generate point and whole-gene mutants and chimeric RVs bearing heterologous NSP3. Previous studies using anti-NSP3 siRNA suggested that NSP3 is dispensable in simian RV replication (153). Use of RG should confirm or refute this theory.

**(e) Sequestration of viral PAMPs by viroplasms.** NSP2 and NSP5—and to a lesser extent, VP2—are the main drivers of viroplasm formation during RV infection. RG studies have shown that NSP5 hyper-phosphorylation and C1Kα-dependent phosphorylation of NSP2 are critical for viroplasm formation (162, 163). These viral reproduction factories may also sequester dsRNA from host sensors with help from additional viral RNA-binding proteins (e.g., NSP3, NSP6) (163). Conversely, viroplasms sequester host antiviral factors, as demonstrated for the IKKε complex and STAT1 by bunyaviruses and MDA5 by human respiratory syncytial virus (164–166). Recent studies have also shown that RV viroplasms specifically exclude host NF-κB, stress granules, and P bodies, blocking antiviral responses (59, 156, 167). Since the dynamic process of viroplasm formation and fusion also involves rearrangement of the cytoskeleton and membranes, these structures may disrupt the trafficking of immunomodulatory host factors while facilitating assembly and trafficking of nascent virions (168, 169). It is also possible that RV viroplasms do not function in innate immunity, similar to influenza A virus cytoplasmic inclusions (170). The RG system will be useful in delineating the relationship between viroplasms, cytoskeleton, and innate immunity.

**(f) Pyroptosis and inflammasome signaling.** Inflammasome activation was recently identified as a contributor to anti-RV host defenses (15, 171, 172). The RNA helicases DHX15 and DHX9 interact with NLRP6 and NLRP9B, respectively, to trigger inflammasome activation in RV-infected murine IECs (15, 171, 172). Blocking this pathway enhances RV replication with concomitant increase in diarrhea prevalence. Furthermore, the cytokines produced by inflammasome activation, IL-1β and IL-18, restrict RV replication and disease in mice, but are produced at low levels in infected humans (136, 173–175). The NLRC4 inflammasome may also contribute to RV restriction via the production of IL-22, a cytokine shown to attenuate RV replication in synergy with IL-18 and type III IFN (176, 177). How RV antagonizes this pathway is not known but can be examined systematically using RG. Studies of other RNA viruses and bacteria indicate antagonism could occur upstream (e.g., parainfluenza virus, Yersinia YopM) or downstream (e.g., enterovirus 71 protease 3C) of inflammasome activation (178–180). These findings will provide the foundation for potential therapeutics targeting inflammasome and pyroptosis to control intestinal inflammation and will be informative to the studies of other enteric pathogens.

**(g) Autophagy and xenophagy.** Autophagy and xenophagy—the degradation of microbial invaders—can either inhibit or facilitate viral pathogenesis (181, 182). Autophagy generally eliminates microbes and provides antigens for lymphocyte recognition, but it can also be usurped to alter viral sensing pathways mediated by RLRs and cGAS-STING (181, 182). Initial studies with simian RV SA11-4F strain suggested that NSP4-mediated release of calcium from the ER activates autophagy via calcium/calmodulin-dependent protein kinase and 5′ AMP-activated kinase pathways, facilitating trafficking of nascent virions (183, 184). Hijacking this pathway relies on the interaction of NSP4-containing COPII vesicles and autophagosome-associated LC3 II (185). Interestingly, autophagosomes fail to fuse with lysosomes in RV-infected cells (184); the viral protein responsible for this inhibition has yet to be identified. A study using RV strains OSU and SA11-4F suggested that autophagy facilitated viral replication (186). RV-encoded small RNAs may also promote autophagy (187, 188). How small RNAs are encoded and processed by viral or cellular factors and how RV impacts autophagy is examinable using RG and will broadly apply to other viruses that avoid lysosomal fusion (189, 190).

**(h) Peroxisome signaling.** Peroxisomes, ubiquitous single-membrane organelles that function in lipid and reactive oxygen species metabolism and signaling, may play critical roles in RV antiviral defense (191–193). Previous studies have demonstrated that peroxisomal MAVS and IRF1-mediated type III IFN pathway restrict viral infection (48, 192–194). Infection with flaviviruses and enteroviruses reduces the number of peroxisomes, and human cytomegalovirus and human herpesvirus-1 infection impedes peroxisomal MAVS signaling (193, 195). On the other hand, peroxisome metabolism is coopted by HIV-1 to facilitate infection (196, 197).

Peroxisomal MAVS alone is sufficient to stimulate robust type I and III IFN in RV-susceptible HT-29 cells (29). Interestingly, the C-terminal VP5* fragment of VP4 in many RV strains (e.g., SA11) harbors a C-terminal tripeptide CRL sequence analogous to canonical peroxisome-targeting sequences (191). Whether VP4 interacts with and impairs peroxisomal MAVS-IRF1 signaling warrants investigation with RG. The potentially redundant or complementary role of VP4 in addition to NSP1 and VP3 in blocking IFN expression should also be investigated with RG to further understanding of potential peroxisome perturbation.

**(i) Other mechanisms of innate immune evasion.** The initial plasmid-only RG system provided the first characterization of NSP6 function in the context of RV infection (198). Previous studies demonstrated that NSP6 localizes to viroplasms and mitochondria, interacting with NSP5 as well as RNA, suggesting a role for NSP6 in RNA binding and viroplasm formation (4, 82, 199–204). Many tissue-culture adapted RV strains (e.g., lapine Alabama, porcine OSU) express truncated NSP6 or lack NSP6 expression altogether. Mattion et al. demonstrated that NSP6 is expressed at low levels in infected cells and plays an expendable role in cell culture (4). For many RNA viruses (influenza A virus NS1, Rift Valley fever virus NSs), these accessory proteins play an instrumental role in innate immune evasion *in vivo* (205, 206). Determining the impact of NSP6 in RV strains which encode a functional NSP6 (e.g., murine EW) will be a critical application of RG to help understand NSP6 in RV replication and pathogenesis *in vivo* (207–209).

NSP1 may have additional IFN-independent roles. Studies using plasmid-only RG systems suggest that NSP1 is dispensable for viral replication since nearly complete replacement with a luciferase reporter negligibly affects viral replication (87, 95). A recent study generated an NSP1-null RV via RG and showed that loss of NSP1 attenuated infection *in vivo* but not *in vitro* (210). *In vivo* attenuation could not be fully rescued in *STAT1* knockout mice, suggesting a previously unknown role for NSP1 in IFN-independent signaling. NSP1 also seems to localize to the nucleus and disrupt promyelocytic nuclear bodies (211). Dissecting the complex activities and associated domains of the multifunctional NSP1 will be challenging, but key to a complete picture of RV innate immune evasion.

## CONSIDERATIONS, DIRECTIONS, AND CONCLUSIONS

The RV RG system, although still in its infancy, has already greatly improved our understanding of RV replication, immunity, and pathogenesis. In this review, we summarized our current knowledge of intensively studied viral antagonists (e.g., NSP1, VP3, NSP2, NSP3) and the potential roles for the RG system in deriving new information on RV and host innate immune evasion (Fig. 1). While several VP4 and VP7 monoreassortants have been generated, including those derived from CDC-9 (G1P[8]), Hosokawa (G4P[8]), KU (G4P[8]), L26 (G12P[4]), Odelia (G4P[8]), WI61 (G9P[8]), and other circulating strains from Africa, the RG system can be leveraged to facilitate the rational design and recovery of recombinant RVs for vaccine purposes (108, 111, 212, 213). Given the low recovery of VP4 and VP7 dual reassortants, additional improvements to the RG system may be desirable. Alternatives to reassorted vaccines include viruses that encode truncated and/or mutated viral proteins, which are expected to yield attenuated strains that replicate at levels low enough to induce immunity without adverse side effects. Most recently, the RG system has been used to engineer portions of the SARS-CoV-2 spike protein into a simian RV SA11 backbone, as a potential combined vaccine for children (214). In the future, a robust RG system may facilitate the creation of bi-, tri-, or multivalent RV vaccines encoding antigens from other enteric viruses (e.g., norovirus, astrovirus, enteric adenovirus), bacteria (enteropathogenic Escherichia coli, *Shigella*), and parasites (Cryptosporidium parvum, Entamoeba histolytica) to maximize vaccination breadth in infants and young children.

The RV RG system also holds great promise and potential for insights into many aspects of basic RV biology. The roles of innate immune evasion in specific steps of RV pathogenesis, including intraintestinal replication, spread to systemic organs, and transmission to naive individuals, are yet to be defined. New and exciting technological advances in molecular biology also give rise to great opportunities to dissect RV-host interactions. Leveraging transposon or random PCR mutagenesis and next-generation sequencing-based phenotypic screens will likely yield new knowledge of how specific RV gene products function in RV replication and immune antagonism. Finally, using RG systems to recover and compare ancient and contemporary RV strains will empower parallel studies of the evolution of host innate immunity and viral innate immune evasion.
